# Synergistic Phytochemical and Pharmacological Actions of Hair Rise^TM^ Microemulsion: A Novel Herbal Formulation for Androgenetic Alopecia and Hair Growth Stimulation

**DOI:** 10.3390/plants13192802

**Published:** 2024-10-06

**Authors:** Anurak Muangsanguan, Warintorn Ruksiriwanich, Pichchapa Linsaenkart, Pensak Jantrawut, Pornchai Rachtanapun, Kittisak Jantanasakulwong, Sarana Rose Sommano, Korawan Sringarm, Chaiwat Arjin, Mathukorn Sainakham, Juan M. Castagnini

**Affiliations:** 1 Department of Pharmaceutical Sciences, Faculty of Pharmacy, Chiang Mai University, Chiang Mai 50200, Thailand; anurak_m@cmu.ac.th (A.M.); pichchapa_li@cmu.ac.th (P.L.); pensak.j@cmu.ac.th (P.J.); mathukorn.s@cmu.ac.th (M.S.); 2 Cluster of Valorization and Bio-Green Transformation for Translation Research Innovation of Raw Materials and Products, Chiang Mai University, Chiang Mai 50200, Thailand; sarana.s@cmu.ac.th (S.R.S.); korawan.s@cmu.ac.th (K.S.); 3 Center of Excellence in Agro Bio-Circular-Green Industry (Agro BCG), Agro-Industry, Chiang Mai University, Chiang Mai 50100, Thailand; pornchai.r@cmu.ac.th (P.R.); kittisak.jan@cmu.ac.th (K.J.); 4 School of Agro-Industry, Faculty of Agro-Industry, Chiang Mai University, Chiang Mai 50100, Thailand; 5 Department of Plant and Soil Sciences, Faculty of Agriculture, Chiang Mai University, Chiang Mai 50200, Thailand; 6 Department of Animal and Aquatic Sciences, Faculty of Agriculture, Chiang Mai University, Chiang Mai 50200, Thailand; chaiwat.arjin@cmu.ac.th; 7 Research Group in Innovative Technologies for Sustainable Food (ALISOST), Department of Preventive Medicine and Public Health, Food Science, Toxicology and Forensic Medicine, Faculty of Pharmacy, Universitat de València, Avenida Vicent Andrés Estellés s/n, 46100 Burjassot, Spain; juan.castagnini@uv.es

**Keywords:** rice bran (*Oryza sativa* L.), shallot bulb (*Allium ascalonicum* L.), licorice root (*Glycyrrhiza glabra* L.), corn kernels (*Zea mays* L.), androgenetic alopecia, Sonic Hedgehog, Wnt/β-catenin, angiogenesis, 5*α*-reductase inhibition

## Abstract

Androgenetic alopecia (AGA) is a genetic condition characterized by an excessive response to androgens, leading to hairline regression in men and hair thinning at the vertex in women, which can negatively impact self-esteem. Conventional synthetic treatments for AGA are often limited by their side effects. In contrast, Thai medicinal plants offer a promising alternative with fewer adverse effects. This study investigates the synergistic phytochemical and pharmacological effects of a novel Hair Rise^TM^ microemulsion, formulated with bioactive extracts from rice bran (*Oryza sativa*), shallot bulb (*Allium ascalonicum*), licorice root (*Glycyrrhiza glabra*), and corn kernels (*Zea mays*), for the treatment of hair loss. The microemulsion, in concentrations of 50%, 75%, and 100% (*v*/*v*), significantly enhanced the proliferation of human hair follicle dermal papilla cells (HFDPCs) compared to minoxidil. Additionally, it upregulated critical hair growth signaling pathways, including Wnt/β-catenin (*CTNNB1*), Sonic Hedgehog (*SHH*, *SMO*, *GLI1*), and vascular endothelial growth factor (*VEGF*), surpassing standard controls such as minoxidil and purmorphamine. The microemulsion also demonstrated potent anti-inflammatory and antioxidant properties by reducing nitric oxide production and oxidative stress, factors that contribute to inflammation and follicular damage in AGA. Furthermore, Hair Rise^TM^ inhibited 5*α*-reductase (types 1–3), a key enzyme involved in androgen metabolism, in both human prostate cancer cells (DU-145) and HFDPCs. These findings suggest that Hair Rise^TM^ microemulsion presents a promising natural therapy for promoting hair growth and reducing hair loss via multiple synergistic mechanisms, offering a potent, plant-based alternative to synthetic treatments.

## 1. Introduction

Androgenetic alopecia (AGA), also known as male and female pattern hair loss, is the most common type, characterized by progressive terminal hair loss after middle age [1]. In general, the major phase of hair follicle formation is composed of the stages of growth (anagen), regression (catagen), resting (telogen), and shedding (exogen), influenced by numerous factors [2]. Indeed, the major common risk in AGA is an androgen hormone. Dihydrotestosterone (DHT) is an androgenic steroid hormone induced by the action of 5*α*-reductase (SRD5A) enzymes, which convert testosterone (TT) to DHT. The overproduction of DHT in androgen-dependent areas may reduce the growth stage in the hair cycle, leading to the shrinkage of the hair follicle and finally hair loss [3]. In addition, inflammation in hair follicles is recognized as a contributing factor in the development of AGA [4]. Oxidative stress and DHT have been shown to elevate nitric oxide (NO) levels in hair follicles [5].

In contrast, the formation of hair follicles requires interaction between the mesenchymal cells and epithelial cells, which receive signaling from human hair follicle dermal papilla cells (HFDPCs), including Wnt/β-catenin (CTNNB1), Sonic Hedgehog (SHH, SMO, and GLI1), and vascular endothelial growth factor (VEGF) signaling pathways [6]. The CTNNB1 signaling pathway is a key pathway for hair growth formation. The stimulation of β-catenin in HFDPCs at the hair germ and hair bulge initiates the lymphoid enhancer factor (LEF) or T-cell factor (TCF) complex and initiates the transcription of downstream target genes such as c-Myc and cyclin D1. This process stimulates the proliferation, migration, and differentiation of HFDPCs in the early stages of the anagen phase [7]. Moreover, CTNNB1 acts as an upstream regulator of the Sonic Hedgehog signaling pathway, which stimulates the development of hair follicles in the middle stage of the anagen stage by promoting the proliferation and migration of epithelial and mesenchymal cells [7,8]. In addition, VEGF supports angiogenesis in the growth stage, enhancing the delivery of nutrient- and oxygen-rich blood to the hair follicles [9].

The FDA-approved treatments for hair loss include topical minoxidil, oral finasteride, and low-level laser therapy [10]. Minoxidil is converted into its active form (minoxidil sulfate), which activates adenosine triphosphate-sensitive potassium (K_ATP_) channels, leading K_ATP_ channels to open. This mechanism makes minoxidil a K_ATP_ channel opener, leading to hyperpolarization of cell membranes, dilation of vascular smooth muscles, and increased blood circulation. The opening of K_ATP_ channels stimulates the growth of HFDPCs, contributing to hair follicle development and stimulating new hair [11]. Finasteride is an inhibitor of the SRD5A type 2 isoenzyme, which was developed to treat benign prostatic hyperplasia and is also used to treat AGA [12]. Furthermore, several treatments, including cell-based treatments, laser therapy, and natural cosmetic product treatments, are being used to treat AGA. The development of novel green cosmetic products containing phytochemicals from natural sources has significantly increased because they are less toxic and more environmentally friendly than conventional cosmetics.

Plants generate constitutive metabolites known as phytochemicals, which are essential to their survival and functionality. Phytochemicals are classified into various categories, such as polyphenols (including phenolic and flavonoid compounds), alkaloids, terpenoids, oryzanols, and tocopherols, each exhibiting diverse biochemical and biological activities based on their chemical structures. [13]. Furthermore, various studies have highlighted the potential of phytochemicals as agents against hair loss by activating hair growth factors such as CTNNB1, Hedgehog, and VEGF, and inhibiting hair loss stimulators like SRD5A, transforming growth factor (TGF-β1), and Dickkopf-related protein 1 (DKK-1) [14]. Rice, also known as *Oryza sativa* L., is extensively consumed on a global scale. The composition of rice grains consists of rice husk and rice bran. Rice bran is the most common by-product of rice milling, accounting for approximately 20% of the total weight of whole rice [15]. Previous studies have indicated that BB3-CMU and BB4-CMU rice bran, which is cultivated in northern Thailand, contains a variety of minerals and bioactive compounds, such as polyphenols (phenolic and flavonoid compounds), oryzanols, and tocopherols [15]. Additionally, shallot bulbs (*Allium ascalonicum* L.) are an economically essential herb in Thailand and are used as a diet and traditional medicine in Asia. Shallot was used for its various pharmacological properties, such as antimicrobial, anticancer, anti-inflammatory, and antioxidant effects [16]. Our previous study showed that shallot extracts contained a high level of polyphenols, including *p*-coumaric acid, quercetin, and rosmarinic acid. Furthermore, our study confirmed the potential of rice bran and shallot extract in terms of their anti-inflammatory activities and their modulation of *SRD5A* gene expression [16,17,18]. Licorice root (*Glycyrrhiza glabra* L.) has been widely used in traditional medicine for centuries, particularly in Asia, including India and China [19]. It contains bioactive compounds such as polyphenols, chromenes, coumarins, and glycyrrhizic acid, with polyphenols and glycyrrhizic acid being the major constituents [20]. Glycyrrhizic acid promotes hair growth by enhancing scalp conditions through its anti-inflammatory properties, which indirectly support hair growth [21]. Zhang et al. reported that glycyrrhizic acid in licorice root could induce the stimulation of hair growth factors and angiogenesis activities in HFDPCs, resulting in hair follicle development and finally hair growth [22]. Additionally, glycyrrhizic acid has been shown to inhibit dihydrotestosterone (DHT) production by suppressing the expression of *SRD5A* genes [21]. Guava (*Psidium guajava* L.) is used as food and traditional medicine due to its pharmacological properties [23]. It is rich in bioactive compounds, particularly polyphenols, which contribute to its antioxidant and anti-inflammatory activities. In Southwest Asia, guava has traditionally been used to stimulate hair growth [23]. Additionally, our previous study demonstrated that guava leaf extract can inhibit the expression of the *SRD5A* gene, which is associated with hair loss [24]. Lastly, corn kernels (*Zea mays* L.), are an essential source of nutrition for humans and animals worldwide. It has been widely used in traditional herbal medicine to treat various diseases, including obesity, edema, and gout [25]. Corn kernels are rich in a variety of phytochemicals, including polyphenols, polysaccharides, proteins, vitamins, and alkaloids, and particularly ferulic acid, which has beneficial antioxidant and anti-inflammatory effects in hair development [26]. All extracts were prepared in the form of microemulsions to increase the stability and skin penetration efficiency of active ingredients. Microemulsion is a drug delivery system composed of water and oil phases stabilized by a combination of surfactant and cosurfactant. The particle size of the microemulsion is less than 100 nm, which achieves optimum effects and offers advantages such as temperature stability, translucency, and long-term settling [27].

As previously mentioned, the bioactive compounds in selected medicinal plant extracts with high potential biological activities were formulated as a multipurpose hair tonic microemulsion, commercially named ‘Hair Rise^TM^’. Therefore, this study aims to estimate the potential of a Hair Rise^TM^ microemulsion containing medicinal plant extracts for treating hair loss and promoting hair growth. The antioxidant, anti-inflammatory, and gene expression regulation involving androgen, Wnt/β-catenin, Sonic Hedgehog, and angiogenesis signaling pathways for AGA treatment of Hair Rise^TM^ microemulsion were determined.

## 2. Results and Discussion

### 2.1. Extraction Yield and Bioactive Compound Estimation

For the preparation of the BB3-CMU rice bran and shallot extracts by ScCO_2_, the extraction conditions were set at a fixed co-solvent, pressure, temperature, and time: 95% (*v*/*v*) ethanol, 400 bar, 50 °C, and 0.5 h, respectively. These conditions were established based on findings from our previous research [28]. Our previous study revealed that high pressure (400 bar) significantly enhances extraction yield compared to lower pressures because the pressure influences the density of carbon dioxide. Increased pressure raises the carbon dioxide density within the extraction column, enhancing the penetration of carbon dioxide and any co-solvents through the material. As a result, this increased density leads to better extraction performance and higher extraction yields [28]. Furthermore, the addition of polar modifiers, such as 95% (*v*/*v*) ethanol as co-solvents in the ScCO_2_ process, could increase the solubility of target compounds in the mixture, thereby improving extraction yields simultaneously [28]. The extraction temperature was fixed at 50 °C because of concerns about the stability of bioactive constituents in the extract. As the temperature increased between 40 and 60 °C, the temperature at 50 °C showed the highest bioactive constituent content [29]. In this study, the BB3-CMU rice bran extracts have a dark green-brown semisolid and greasy texture, with an extraction yield of 14.80 ± 0.04% (*w*/*w*) based on the dry materials (Table 1). The BB3-CMU rice bran extract showed the highest content of polyphenols (32.42 ± 1.42 mg GAE/g extract for total phenolic content and 56.03 ± 1.14 mg EGCG/g extract for total flavonoid content). These results align with previous studies indicating that BB3-CMU rice bran showed high contents of polyphenols [15]. The successful extraction of these compounds from BB3-CMU rice bran is influenced by various factors, such as extraction temperature, co-solvent type, pressure, and degree of mechanical agitation [30]. These factors contribute to achieving a greater concentration of bioactive compounds in the extracts. The three main bioactive compounds found in BB3-CMU rice bran were *α*-tocopherol (8.54 ± 0.02 mg/g extract), *γ*-tocopherol (3.65 ± 0.01 mg/g extract), and chlorogenic acid (0.11 ± 0.01 mg/g extract). Our results were consistent with previously established findings that tocopherol was primarily evident in rice bran [18]. A recent study reported that tocopherol could induce the expression of genes related to hair growth, especially Wnt/β-catenin activity in HFDPCs. This activation contributes to an extended growth phase in the hair cycle and encourages new hair development [17]. Chlorogenic acid has been reported to be the main phenolic compound in rice bran, rice husk, and grain [15]. Furthermore, chlorogenic acid has been reported to have anti-hair loss activities via the anti-androgen pathway by inhibiting the expression of the *SRD5A* gene and stimulating new hair via the inducer growth factors’ gene expression in the hair follicle formation process [28]. In addition, a previous study indicated that unsaturated fatty acids (oleic acid and *γ*-linolenic acid) found in rice bran extract significantly suppressed *SRD5A* expression in HFDPCs [2].

The shallot extract appeared greasy, dark gray, and semisolid. The extraction yield was 5.85 ± 0.09% (*w*/*w*) based on dry material The total phenolic and flavonoid content of shallot was 4.18 ± 0.03 mg GAE/g extract and 2.54 ± 0.01 mg EGCG/g extract, respectively (Table 1). The phenolic compounds found in shallot extract were *p*-coumaric acid (1.02 ± 0.01 mg/g extract), followed by rosmarinic acid (0.21 ± 0.01 mg/g extract), and quercetin (0.03 ± 0.01 mg/g extract). Our results were consistent with previous findings that shallot extract contains *p*-coumaric acid [31]. Moreover, Khantham et al. reported the potential of shallot extracts for their beneficial effects on the stimulation of hair growth, including anti-androgenic and anti-inflammation pathways [16].

The corn kernels and licorice root were extracted by conventional maceration (CE). CE is a widely used method for extracting bioactive compounds with recognized biological activities from medicinal plants [32]. In this study, the extraction yield of corn kernels (22.41 ± 0.86% (*w*/*w*) based on dry material) was higher than that of licorice root (11.21 ± 0.24% (*w*/*w*) based on dry material) (Table 1). The physical appearance of corn kernel extract was dark yellow, semisolid, and greasy. Licorice root is dark brown, semisolid, and greasy. The phenolic and flavonoid compounds of licorice root extract were 3.21 ± 0.12 mg GAE/g extract and 1.96 ± 0.08 mg EGCG/g extract, respectively. The primary bioactive compound in licorice root was glycyrrhizic acid (0.62 ± 0.02 mg/g extract). Glycyrrhizic acid is recognized for its steroid-like properties and strong pharmacological actions, such as anti-inflammatory, anti-gastric ulcer, anti-hepatotoxic, and antiviral activities [33]. In addition, Zhang et al. reported the potential of glycyrrhizic acid at different concentrations (1%, 10%, and 20% (*v*/*v*) in water) on hair growth in mice compared to minoxidil, a standard treatment for anti-hair loss. The results showed that glycyrrhizic acid at 10% and 20% (*v*/*v*) in water stimulated hair density in mice higher than minoxidil [22]. Moreover, glycyrrhizic acid enhanced the expression of Wnt/β-catenin and angiogenesis activities in HFDPCs, higher than minoxidil [22]. Lastly, the phenolic and flavonoid contents of the corn kernel extract were 14.42 ± 0.56 mg GAE/g extract and 4.33 ± 0.12 mg EGCG/g extract, respectively. Ferulic acid (5.14 ± 0.01 mg/g extract), chlorogenic acid (4.22 ± 0.51 mg/g extract), and *p*-coumaric acid (0.33 ± 0.01) were detected. Ferulic acid has been reported to exhibit various pharmacological activities, particularly antioxidant and anti-inflammatory effects in the hair cycle [26]. Furthermore, Xu et al. reported the potential of ferulic acid to enhance mRNA expression in DHT-irritated HFDPCs and stimulate hair growth in mice with androgenetic alopecia [34].

### 2.2. Antioxidant Activities of Plant Extracts

The antioxidant properties of all plant extracts were evaluated using the ABTS and DPPH scavenging assays (Table 1). Among the tested extracts, the BB3-CMU rice bran extract exhibited the strongest antioxidant activity, with values of 32.14 ± 2.03% and 28.89 ± 1.08% for ABTS and DPPH radicals, respectively. The high level of polyphenol compounds significantly contributes to their strong antioxidant activities [35]. Polyphenols are known for their antioxidant properties due to a high number of hydroxyl groups in their structures [36]. Therefore, BB3-CMU rice bran not only contained the highest polyphenol content, but also demonstrated superior efficacy in neutralizing ABTS and DPPH radicals.

As previously described, all plant extracts demonstrated high levels of bioactive compounds. These compounds can stimulate hair growth through various pathways, including antioxidant, anti-inflammatory, and anti-androgenetic pathways, which prolong the duration of the anagen stage of the hair cycle. Additionally, these compounds can activate hair growth pathways such as Wnt/β-catenin, Sonic Hedgehog, and angiogenesis pathways, which support hair growth through prolonging the anagen stage and stimulating blood flow to the hair follicles. Based on our data, all plant extracts were selected for the development of a Hair Rise^TM^ microemulsion formulation.

### 2.3. Characterization of the Hair Rise^TM^ Microemulsion

The physical stability of the Hair Rise^TM^ microemulsion was determined by observing changes in particle size, or Z-average (Z-Ave), polydispersity index (pdI), and zeta potential (ZP) values after 3 months of storage at room temperature. After 3 months of storage, the Z-Ave of Hair Rise^TM^ microemulsion at initial, 1, 2, and 3 months demonstrated values of 30.57 ± 0.17, 32.43 ± 0.06, 38.96 ± 1.32, and 48.38 ± 0.88 nm, respectively. Moreover, after six cycles of heating–cooling acceleration, the Z-Ave showed a value of 42.43 ± 0.91 nm. Microemulsions are small colloidal dispersion systems with particle sizes ranging from 10 to 100 nm. The small particle size could provide advantages in the Hair Rise^TM^ formulation, such as thermal stability, translucency, and long-term stability. Furthermore, pdI, defined as the ratio of the standard deviation to the average particle size, indicates the uniformity of the particle sizes. The low pdI value (<0.4 nm) demonstrates the high uniformity of the particle sizes in the Hair Rise^TM^ formulation [37]. 

The ZP is a crucial characteristic of particles that influences both their stability and ability to adhere to cells. The ZP, whether positive or negative, plays a vital role in maintaining the stability of particle suspensions. This stability is primarily due to the electrostatic repulsion observed among particles with similar charges, which results in their separation. Our results showed that the Hair Rise^TM^ microemulsion was highly stable when stored for 3 months (Table 2). The high stability of the Hair Rise^TM^ microemulsion indicates that it functions as a thermodynamically stable system [38].

### 2.4. Effect of Hair Complex Microemulsion on Cell Viability and Proliferation

The effects of plant extracts and Hair Rise^TM^ microemulsions on cell viability and proliferation were assessed across concentrations from 0.0625 to 2 mg/mL in RAW 264.7, DU-145, and HFDPCs. According to ISO 10993-5 guidelines, a cell survival rate exceeding 80% is regarded as non-toxic [39]. After 24 h of treatments, plant extracts (BB3-CMU rice bran, shallot bulb, licorice root, and corn kernels) at a concentration ≥ 0.5 mg/mL demonstrated cytotoxicity and significantly decreased the viability of all types of cells compared to untreated cells. The maximum concentration of plant extract (0.25 mg/mL) that maintained cell viability above 80% was classified as non-toxic.

**Figure 1:**
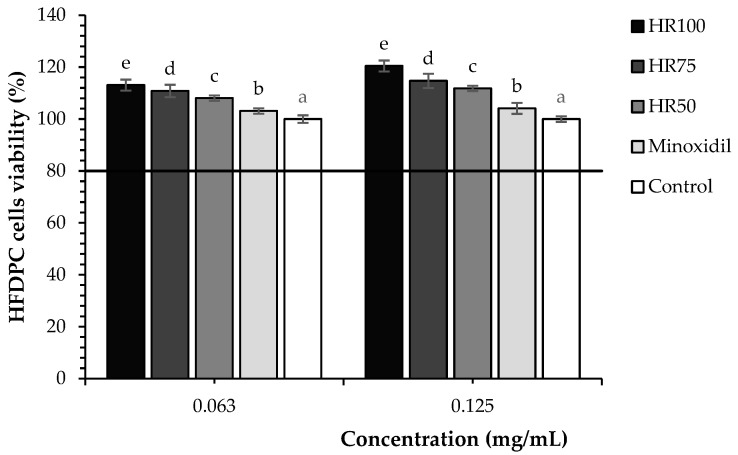
The effect of Hair Rise^TM^ microemulsions and standard controls (minoxidil) at concentrations of 0.063 and 0.125 mg/mL on the proliferation of HFDPCs was compared to untreated cells. Values were presented as the mean ± SD for measurements for each group. A one-way ANOVA was used for statistical analysis, followed by Tukey’s HSD test. Statistical differences (*p* < 0.05) in cell proliferation among samples at each treatment concentration (0.063 and 0.125 mg/mL) are indicated by different letters (a–e). HR100: 100% of Hair Rise^TM^ microemulsion; HR75: Hair Rise^TM^ microemulsion at a concentration of 75% (*v*/*v*) in water; HR50: Hair Rise^TM^ microemulsion at a concentration of 50% (*v*/*v*) in water.

In the formulation part, all concentration (50, 75, and 100% (*v*/*v*) in water) of Hair Rise^TM^ microemulsion at a concentrations ≥ 0.5 mg/mL in all cells showed no cytotoxicity at the comparable level of untreated cells (*p* > 0.01). So, the highest concentration of 0.5 mg/mL, which gave viability above 80%, was selected for further experiments. Furthermore, all of the different concentrations of Hair Rise^TM^ microemulsion significantly increased the proliferation of HFDPCs at concentrations of 0.063 and 0.125 mg/mL compared with the untreated cells (*p* > 0.01) (Figure 1). Moreover, the proliferation of HFDPCs at all concentrations (50, 75, and 100% (*v*/*v*) in water) of Hair Rise^TM^ microemulsion was higher than that of minoxidil. As is known, minoxidil could promote the cell viability of HFDPCs by activating the ERK and AKT signaling pathways, preventing cell death, and increasing the cell viability of HFDPCs [40,41]. 

HFDPCs play a vital role in hair follicle formation and the stimulation of hair growth [42]. The process of hair follicle formation consists of three primary stages: induction, organogenesis, and cytodifferentiation, and these three stages include eight steps [43]. Firstly, HFDPCs generate signaling for hair follicle cell regeneration, which induces the proliferation, migration, and differentiation of epithelial cells, finally forming the hair follicle basal plate. After that, the hair follicle basal plate sends the signal back to the HFDPCs to stimulate the aggregate of HFDPCs and lead to the formation of the new hair follicle. In the second step, aggregation of the HFDPCs generates the signaling to induce the expansion of the hair follicle basal plate downward to enter the dermis and form the hair buds. Then, the hair buds expand and elongate in the dermis, and the fibroblast cells in the dermis translocate to the hair buds. Afterward (third step), HFDPCs induce the proliferation of keratinocyte cells. These keratinocyte cells overlap and form a column around the hair buds. In the fourth step, HFDPCs under the hair follicle basal plate translocate to the hair follicle basal plate and form dermal papillae. The fourth and the fifth steps occur at the same time; HFDPCs induce the proliferation and migration of the hair matrix, leading to the differentiation of hair matrix cells to the transit-amplifying progenitor cells and the formation of the hair shaft and inner root sheath, respectively. In the sixth step, the accessory organ of the hair follicle structure is completely developed by the formation of epithelial cells. In the seventh step, the hair follicle elongates and moves through the hair canal. Finally (eighth step), the hair follicle is completely formed, and the hair shaft enters through the epidermis [43,44,45].

As previously described, HFDPCs play an essential role in all steps of hair follicle development. This study indicated that all Hair Rise^TM^ microemulsion samples at concentrations of 0.063 and 0.125 mg/mL promoted HFDPC proliferation, which may promote the growth of hair follicles, leading to hair growth stimulation [42]. Moreover, high concentrations of Hair Rise^TM^ microemulsion showed greater HFDPC proliferation compared to lower concentrations in the cell proliferation assay (0.063 and 0.125 mg/mL). These results demonstrate that a higher concentration of Hair Rise^TM^ microemulsion enhances HFDPC proliferation more effectively than a lower concentration [46]. This result may be attributed to the synergistic effects of compounds present in the Hair Rise^TM^ microemulsion, such as vitamin B5, and the bioactive compounds in plant extracts, including ferulic acid, *α*-tocopherol, and other components. These compounds align with previous studies showing that ferulic acid, *α*-tocopherol, and vitamin B5 can stimulate the proliferation of HFDPCs and promote hair growth. [17,47,48].

### 2.5. Effect of Hair Complex Serum on Anti-Inflammatory Activities

**Figure 2:**
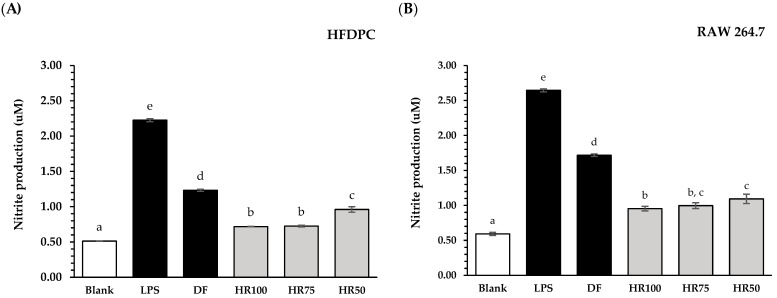
Effects of Hair Rise^TM^ microemulsions and standard controls (diclofenac sodium) at 0.5 mg/mL on nitrite level in the lipopolysaccharide (LPS)-induced HFDPCs (**A**) and RAW 264.7 (**B**) cells for 24 h compared to untreated cells (blank) and LPS-induced control (+LPS). DF: diclofenac sodium; HR100: 100% Hair Rise^TM^ microemulsion; HR75: Hair Rise^TM^ microemulsion at a concentration of 75% (*v*/*v*) in water; HR50: Hair Rise^TM^ microemulsion at a concentration of 50% (*v*/*v*) in water. Values are presented as the mean ± SD for triplicates in each sample. A one-way ANOVA was used for statistical analysis, followed by Tukey’s HSD test. Statistical differences (*p* < 0.05) are represented by different letters (a–e).

Increased NO levels have been reported to impact hair regeneration [4]. The inflammation in hair follicles is induced by oxidative stress and DHT [5]. Previous studies reported that the NO level in HFDPCs of patients with androgenetic alopecia was higher than that of healthy volunteers [49]. In this study, all concentrations (50%, 75%, and 100% (*v*/*v*) in water) of Hair Rise^TM^ microemulsion and the standard drugs (diclofenac sodium) at a concentration of 0.5 mg/mL were evaluated on HFDPCs and RAW 264.7 cells to compare their inhibitory effects on NO production. NO production was induced by lipopolysaccharide (LPS), an inflammatory stimulator derived from *Escherichia coli* [50]. As demonstrated in Figure 2, both in HFDPCs and RAW 264.7 cells, the level of nitrite produced by the LPS-stimulated group without any prior treatment was higher than that of the solvent-treated control group (blank). Moreover, all tested samples significantly suppressed nitrite production compared to the nitrite level of the LPS-stimulated group (*p* < 0.05). As a result, all concentrations (50%, 75%, and 100% (*v*/*v*) in water) of Hair Rise^TM^ microemulsion showed significantly higher nitrite suppression than diclofenac sodium (a standard inflammatory drug) in both HFDPCs and RAW 264.7 cells (*p* < 0.05). In comparison to Hair Rise^TM^ microemulsion at different concentrations (50%, 75%, and 100% (*v*/*v*) in water), there was no significant difference between the nitrite level of 75% (*v*/*v*) in water and 100% of Hair Rise^TM^ microemulsion. Interestingly, the lowest concentration of Hair Rise^TM^ microemulsion (50% (*v*/*v*) in water) showed higher suppression than the diclofenac sodium in both HFDPCs and Raw 264.7 cells. Hair Rise^TM^ microemulsion contains several compounds, including vitamin B5, which comply with previous research indicating that these substances can promote hair growth by suppressing nitrite production in hair follicle cells [51,52]. Furthermore, the bioactive compounds in rice bran, shallot bulb, licorice root, and corn kernels have been reported, such as *α*-tocopherol, *γ*-oryzanol, glycyrrhizic acid, and polyphenols, which could suppress LPS-induced nitrite production [16,17,22,53]. Plant extracts in the formulation of Hair Rise^TM^ microemulsion contain many anti-inflammation compounds that could suppress nitrite production and decrease inflammatory-induced perifollicular damage in hair loss.

### 2.6. Effect of Hair Rise^TM^ Microemulsion on Antioxidant Activities in HFDPCs

**Figure 3:**
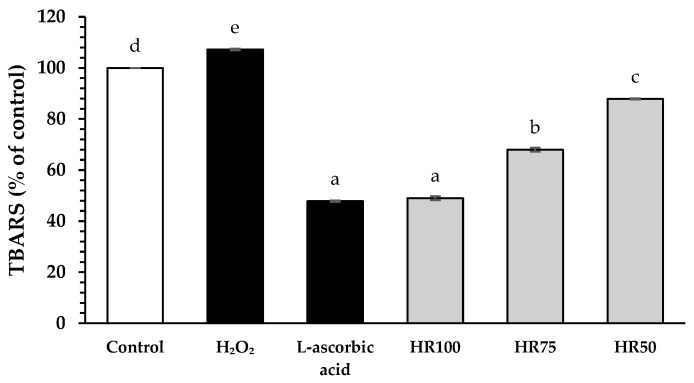
Effects of Hair Rise^TM^ microemulsions and standard controls (L-ascorbic acid) at the concentration of 0.5 mg/mL on the malondialdehyde production in hydrogen peroxide (H_2_O_2_)-induced HFDPCs using the thiobarbituric acid reactive substances (TBARS) assay. HR100: 100% Hair Rise^TM^ microemulsion; HR75: Hair Rise^TM^ microemulsion at a concentration of 75% (*v*/*v*) in water; HR50: Hair Rise^TM^ microemulsion at a concentration of 50% (*v*/*v*) in water. Values are presented as the mean ± SD for triplicates in each sample. A one-way ANOVA was used for statistical analysis, followed by Tukey’s HSD test. Statistical differences (*p* < 0.05) are represented by different letters (a–e).

Malondialdehyde is a by-product generated by the lipid peroxidation process. The reaction between lipid peroxidation and thiobarbituric acid produces a dark pink compound that can be detected by a UV–Vis spectrophotometer [54]. The thiobarbituric acid reactive substances (TBARS) method was used to determine the malondialdehyde production in hydrogen peroxide (H_2_O_2_)-stimulated HFDPCs, as demonstrated in Figure 3. The antioxidant activities of all concentrations (50%, 75%, and 100% (*v*/*v*) in water) of Hair Rise^TM^ microemulsion were compared to the standard control L-ascorbic acid (47.81 ± 0.24% of control) as a positive control. The TBARS level of hydrogen peroxide-induced HFDPCs (107.26 ± 0.32% of control) was significantly different from the untreated cells (control group) (*p* < 0.05). All concentrations (50%, 75%, and 100% (*v*/*v*) in water) of Hair Rise^TM^ microemulsion showed antioxidation activities with decreased TBARS levels at 87.88 ± 0.19%, 68.01 ± 0.74%, and 49.03 ± 0.73% of control, respectively. Interestingly, 100% Hair Rise^TM^ microemulsion showed a comparable TBARS level to L-ascorbic acid (positive control) at *p* < 0.05. A previous study reported that TBARS levels were significantly higher in AGA patients compared to that of the healthy group, whereas SOD and GSH levels were significantly lower in AGA patients than those in the healthy group [55].

Hair Rise^TM^ microemulsion contains several antioxidant substances, such as vitamin B5. Furthermore, vitamin B5 plays an important role in protecting cells from ROS by regulating the synthesis of acetyl coenzyme A, which is important for several cellular processes. Specifically, vitamin B5 can remove lipid peroxidation products, enhance cell membrane repair mechanisms, and synthesize cholesterol [56]. In addition, selected medicinal plant extracts and specific compounds with antioxidant and anti-inflammatory effects play a crucial role in the hair follicle. The bioactive compounds in Hair Rise^TM^ microemulsion play an important role as free radical scavengers, suppressors of radical chain reactions and oxidative enzymes, metal chelators, and stimulators of antioxidant enzymes, including CAT, SOD, and GSH, that could protect the hair follicle against ROS under oxidative stress [57].

### 2.7. Effects of Hair Rise^TM^ Microemulsion on Gene Expression

**Figure 4:**
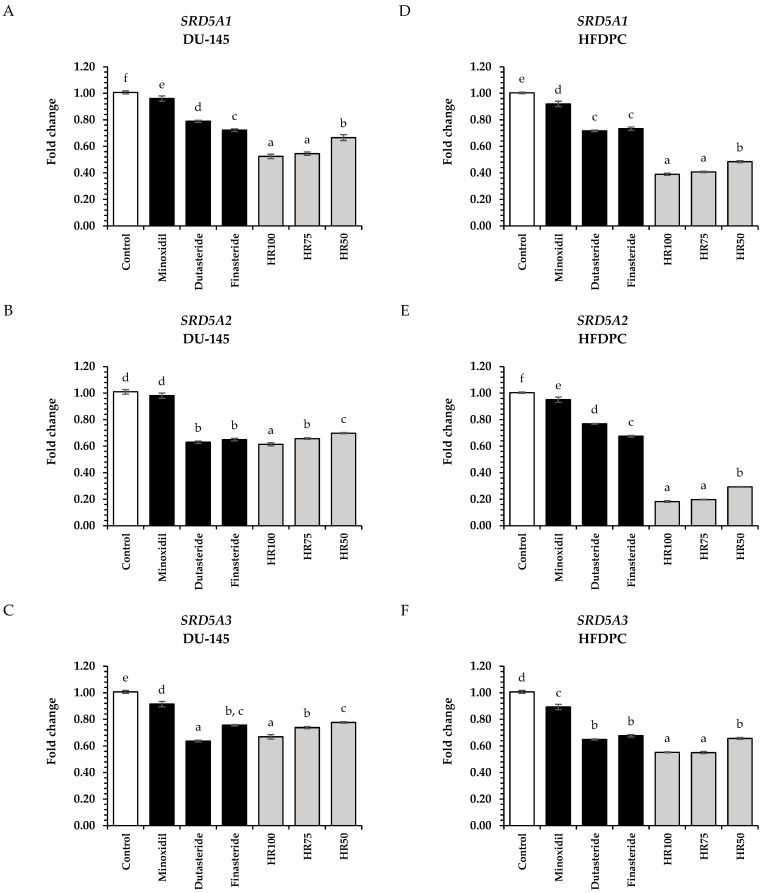
The effects of Hair Rise^TM^ microemulsions on the suppression of genes in the androgen pathway; (**A**) *SRD5A1*, (**B**) *SRD5A2*, and (**C**) *SRD5A3* in DU-145 cells; and (**D**) *SRD5A1*, (**E**) *SRD5A2*, and (**F**) *SRD5A3* in HFDPCs were compared to the standard controls (minoxidil, dutasteride, and finasteride) at a concentration of 0.5 mg/mL. HR100: 100% Hair Rise^TM^ microemulsion; HR75: Hair Rise^TM^ microemulsion at a concentration of 75% (*v*/*v*) in water; HR50: Hair Rise^TM^ microemulsion at a concentration of 50% (*v*/*v*) in water. The results are shown as a fold change in gene expression relative to the untreated cells (control group). A one-way ANOVA was used for statistical analysis, followed by Tukey’s HSD test. Statistical differences (*p* < 0.05) are represented by different letters (a–f).

Hair regeneration is a complex process. Generally, the follicle’s major stages of the hair cycle include growth, regression, resting, and shedding, all influenced by various factors [2]. The main regulatory pathways of hair regeneration, including the androgen, Wnt/β-catenin, Sonic Hedgehog, and angiogenesis signaling pathways, were evaluated in this study [42]. DHT is an androgenic steroid hormone induced by the action of SRD5A enzymes, which convert testosterone to DHT. The overproduction of DHT in androgen-dependent areas may shorten the growth stage in the hair cycle, resulting in the early shrinkage of the hair follicle, and finally hair loss [3]. Hair Rise^TM^ microemulsion contains several compounds, including tocopherol, vitamin B5, ferulic acid, and various plant extracts. Previous research indicated that *α*-tocopherol and *γ*-oryzanol in rice bran played an essential role in SRD5A enzyme inhibition (Table 3) [17,18]. Moreover, shallot extract contains a lot of *p*-coumaric acid, rosmarinic acid, and quercetin, which also suppresses SRD5A activity, similar to glycyrrhizic acid in licorice root [16,58]. Initially, we evaluated the effects of Hair Rise^TM^ microemulsions on the expression of genes associated with the androgen pathway (*SRD5A1*, *SRD5A2*, and *SRD5A3*) in DU-145 cells and HFDPCs. The Hair Rise^TM^ microemulsion and standard controls, including minoxidil, dutasteride, and finasteride, were determined at 0.5 mg/mL. The suppression effects of Hair Rise^TM^ microemulsion on genes associated with the androgen pathway are shown in Figure 4. All of the Hair Rise^TM^ microemulsions significantly inhibited the expression of *SRD5A1*, *SRD5A2*, and *SRD5A3* compared to the untreated cells (control group) and standard controls (minoxidil, dutasteride, and finasteride) in both DU-145 cells and HFDPCs (*p* < 0.05). The 100% Hair Rise^TM^ microemulsion showed the highest *SRD5A1*, *SRD5A2*, and *SRD5A3* gene suppression at 0.52 ± 0.02, 0.61 ± 0.01, and 0.67 ± 0.02 in DU-145 cells and 0.39 ± 0.01, 0.18 ± 0.01, and 0.55 ± 0.01 in HFDPCs, respectively, (*p* < 0.05).

The *SRD5A* gene expression of Hair Rise^TM^ microemulsion demonstrated in a concentration-dependent manner in both DU-145 and HFDPCs. However, the 75% (*v*/*v*) of Hair Rise^TM^ microemulsion showed no significant difference in the *SRD5A* genes suppression compared to the 100% of Hair Rise^TM^ microemulsion in both DU-145 and HFDPCs (*p* > 0.05). Furthermore, the Hair Rise^TM^ microemulsion showed the inhibition of *SRD5A* in HFDPCs, which are key to hair shaft development and play a central role in promoting hair growth, compared to DU-145 cells, a prostate cancer cell line commonly used to study 5*α*-reductase isoenzymes.

HFDPCs are responsible for the formation of hair follicles and the production of hair growth. As previously described, the formation of hair follicles requires interaction between the mesenchymal cells and epithelial cells, which receive signaling from HFDPCs [6]. The Wnt/β-catenin (*CTNNB1*) signaling pathway is central to HFDPC proliferation, where Wnt proteins bind to Frizzled receptors and a low-density lipoprotein-related protein (LRP) or tyrosine kinase receptors, stabilizing β-catenin to prevent degradation. This activates downstream genes responsible for cell proliferation and migration, supporting hair growth [59,60].

The Wnt/β-catenin signaling pathway is an upstream process of the Sonic Hedgehog signaling pathway [7]. The Sonic Hedgehog signaling pathway is pivotal for signal transduction between mesenchymal and epithelial cells and plays a role in intracellular regulation. Finally, this pathway stimulates the development of the epidermis and hair follicles, repairs damage, and maintains the characteristics of hair follicle bulge stem cells. In the canonical Sonic Hedgehog signaling pathway, Sonic Hedgehog (SHH) ligands bind to the transmembrane receptor protein, or Patched (PTCH), which is a repressor of the membrane protein smoothened (SMO). After that, SMO-free interacts with the EVC complex (Ellis van Creveld Syndrome) and translocate to the primary cilia. Then, SMO activates glioma-associated oncogene (GLI) family transcription factors and protein kinase A (PKA) to form a macromolecular complex. Finally, the GLI complex passes through the nucleus to stimulate transcription of downstream target genes, resulting in the acceleration of the transition of telogen to the anagen stage, and enhancing hair follicle development by promoting the proliferation of HFDPCs, mesenchymal, epithelial, and fibroblast cells in the anagen phase [8,61].

Furthermore, vascular endothelial growth factor (VEGF) plays a vital role in promoting angiogenesis during the growth stage of hair follicles. Its function involves enhancing oxygen and nutrient delivery to the follicles, leading to an increase in follicle diameter and stimulating hair growth [62]. The effects of the Hair Rise^TM^ microemulsion on genes such as *CTNNB1*, *SHH*, *SMO*, *GLI1*, and *VEGF*, which are involved in hair growth pathways, were evaluated. In HFDPCs, the Hair Rise^TM^ microemulsion and standard controls, including minoxidil and purmorphamine, were determined at a concentration of 0.5 mg/mL.

**Figure 5:**
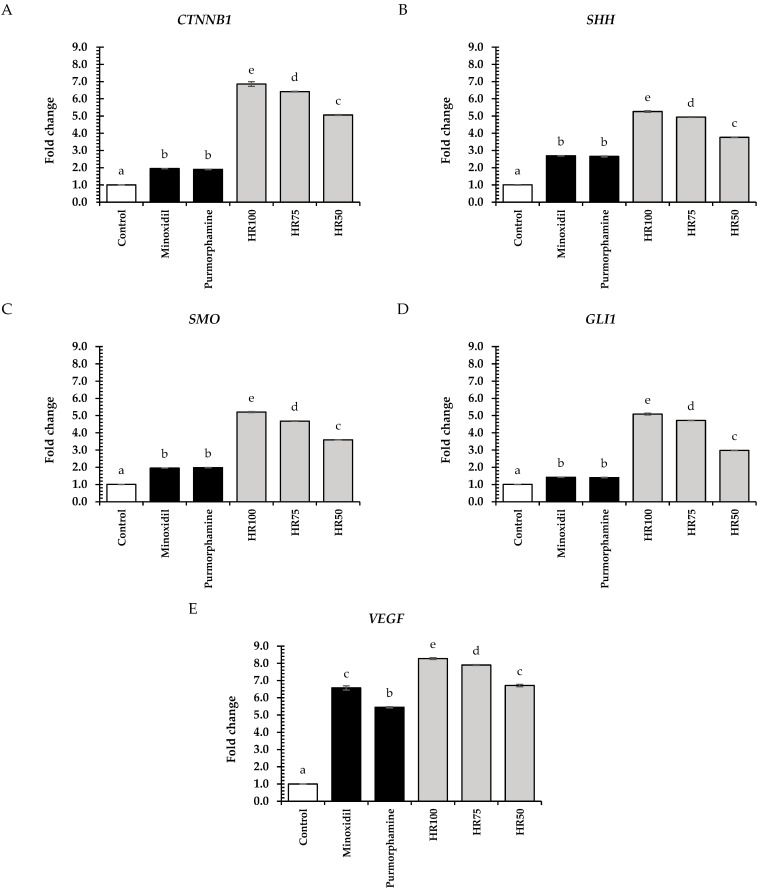
Effects of Hair Rise^TM^ microemulsions and standard control (minoxidil and purmorphamine) (0.5 mg/mL) on the relative mRNA expression of genes associated with Wnt/β-catenin signaling (**A**) *CTNNB1*; Sonic Hedgehog pathways (**B**) *SHH*; (**C**) *SMO*; (**D**); *GLI1*; and angiogenesis (**E**) *VEGF* in HFDPCs. HR100: 100% Hair Rise^TM^ microemulsion; HR75: Hair Rise^TM^ microemulsion at a concentration of 75% (*v*/*v*) in water; HR50: Hair Rise^TM^ microemulsion at a concentration of 50% (*v*/*v*) in water. The results are shown as a fold change in gene expression relative to the untreated cells (control group). A one-way ANOVA was used for statistical analysis, followed by Tukey’s HSD test. Statistical differences (*p* < 0.05) are represented by different letters (a–e).

Considering the stimulation signaling pathway, the Wnt/β-catenin signaling pathway (Figure 5A), and the Sonic Hedgehog signaling pathway (Figure 5B–D), all concentrations of the Hair Rise^TM^ microemulsion significantly stimulated *CTNNB1*, *SHH*, *SMO*, and *GLI1* expression compared to the untreated group and standard controls (minoxidil and purmorphamine) (*p* < 0.05). In the Wnt/β-catenin signaling pathway, the 100% Hair Rise^TM^ microemulsion showed the greatest fold change expression of 6.86 ± 0.14, followed by the 75% and 50% (*v*/*v*) Hair Rise^TM^ microemulsion at 6.42 ± 0.02 and 5.06 ± 0.02, respectively. These results suggest that Hair Rise^TM^ microemulsion could stimulate hair follicle growth, which is regulated by Wnt/β-catenin-related genes. For the Sonic Hedgehog signaling pathway, the 100% Hair Rise^TM^ microemulsion expressed the greatest fold change in all tested genes of *SHH* (5.26 ± 0.04), *SMO* (5.20 ± 0.03), and *GLI1* (5.08 ± 0.05), followed by the 75% and 50% (*v*/*v*) Hair Rise^TM^ microemulsion, respectively. Previous research indicated that chlorogenic acid, epigallocatechin gallate, and quercetin promote the expression of Wnt/β-catenin [16,63,64]. Our results showed that the plant extracts in the Hair Rise^TM^ microemulsion used in this study contain chlorogenic acid, epigallocatechin gallate, and quercetin, which notably increased the accumulation of β-catenin in the cytoplasm of HFDPCs, leading to the transcription of *CTNNB1*. The transcription of *CTNNB1* may support the transition from the telogen to the anagen stage and stimulate hair growth (Table 3) [59]. Furthermore, recent studies have indicated that quercetin in plant extracts promotes hair growth by stimulating the Sonic Hedgehog signaling pathway [65]. Our findings demonstrated that the Hair Rise^TM^ microemulsion, enriched with plant extracts containing quercetin, induced the expression of Sonic Hedgehog-related genes (*SHH*, *SMO*, and *GLI1*) in HFDPCs. These activations may support hair growth by facilitating the transition from the telogen to anagen stage [8]. Additionally, our results demonstrated that the Hair Rise^TM^ microemulsion significantly stimulated the expression of *CTNNB1*, *SHH*, *SMO*, and *GLI1* genes in a concentration-dependent manner. 

All concentrations of the Hair Rise^TM^ microemulsion significantly exhibited higher *VEGF* expression activity compared to the untreated cells (control group) and standard controls (minoxidil and purmorphamine) (*p* < 0.05). Especially, the 100% Hair Rise^TM^ microemulsion showed the highest *VEGF* expression (8.27 ± 0.04), followed by the 75% and 50% (*v*/*v*). This result may be attributed to the combined effects of various compounds in the Hair Rise^TM^ microemulsion, including vitamin B5. Previous research shows that vitamin B5 can stimulate *VEGF* expression and induce wound healing [56]. Furthermore, our results demonstrated that all concentrations of the Hair Rise^TM^ microemulsions showed higher activity than the standard control, especially minoxidil, which is a well-known VEGF stimulator that could support angiogenesis during active hair regeneration [11].

**Table 3:** Mechanisms of medicinal plant extracts for hair growth promotion and hair loss prevention.

Plant Material	Bioactive Compound	Mechanism	Associated Biomarker	Reference
Rice bran	Chlorogenic acid *p*-Coumaric acid Rosmarinic acid Ferulic acid Epigallocatechin gallate Quercetin *α*-Tocopherol *γ*-Tocopherol	Hair growth stimulation	Wnt/β-catenin ↑ Sonic Hedgehog ↑ Angiogenesis ↑ Growth factor (*IGF-1*, *KGF*) ↑	[2,66]
		Hair loss prevention	5*α*-reductase ↓ Inflammatio*n* ↓ Oxidative stress ↓	[2,66,67]
Shallot bulb	*p*-Coumaric acid Rosmarinic acid Quercetin	Hair growth stimulation	Wnt/β-catenin ↑ Sonic Hedgehog ↑ Angiogenesis ↑ Growth factor (*IGF-1*) ↑ Hair follicle proliferation ↑	[16,68]
		Hair loss prevention	5*α*-reductase ↓ Inflammatio*n* ↓ Oxidative stress ↓	[16,69]
Licorice root	Chlorogenic acid Epigallocatechin gallate Quercetin	Hair growth stimulation	Hair follicle proliferation ↑	[21]
		Hair loss prevention	5*α*-reductase ↓ Inflammatio*n* ↓ Oxidative stress ↓	[21,70,71]
Corn kernels	Chlorogenic acid *p*-Coumaric acid Glycyrrhizic acid	Hair loss prevention	Inflammatio*n* ↓ Oxidative stress ↓	[72,73]

Note: IGF-1: insulin-like growth factor-1; KGF: keratinocyte growth factor.

## 3. Materials and Methods

### 3.1. Chemicals and Reagents

PEG-40 hydrogenated castor oil, Tween 20, Tween 80, and Propylene glycol were purchased from NSG Namsiang (Namsiang Co., Ltd., Chiang Mai, Thailand), while the following substances were obtained from MySkinRecipes (Chanjao Longevity Co., Ltd., Bangkok, Thailand): vitamin B5, disodium EDTA, and sodium hydroxide. The Folin–Ciocalteu reagent, Triton X-100, aluminum chloride hexahydrate, and hydrogen peroxide were purchased from Merck (Darmstadt, Germany). The 2,2′-azino-bis (ethylbenzthiazoline-6-sulfonic acid) (ABTS), 2,2-diphenyl-1-picrylhydrazyl (DPPH), epigallocatechin gallate (EGCG), gallic acid, 6-hydroxy-2,5,7,8-tetramethylchroman-2-carboxylic acid (Trolox), sulforhodamine B (SRB), diclofenac sodium, L-ascorbic acid, and dimethyl sulfoxide (DMSO) were sourced from Sigma Chemical (St. Louis, MO, USA). Thiobarbituric acid was obtained from VWR Chemicals (BDH Chem. Ltd., Poole, UK). Furthermore, finasteride, dutasteride, and minoxidil were sourced from Wuhan W&Z Biotech (Wuhan, China). For cell culture, the Dermal Papilla Growth Medium was purchased from Promo Cell GmbH (Heidelberg, Germany), while Roswell Park Memorial Institute 1640 Medium (RPMI-1640), Dulbecco’s Modified Eagle Medium (DMEM), Fetal bovine serum (FBS), antibiotic-antimycotic (100×), and penicillin/streptomycin (100×) solutions were sourced from Gibco Life Technologies (Thermo Fisher Scientific, Waltham, MA, USA). Griess reaction colorimetric kit was sourced from Invitrogen (Thermo Fisher Scientific, Inc., Eugene, OR, USA). All other chemicals used in this study were of analytical grade.

### 3.2. Plant Materials and Preparation of Extract

The bran of *Oryza sativa* cv. Bue Bang 3-CMU (BB3-CMU) was acquired from the Lanna Rice Research Center, Chiang Mai University, Chiang Mai, Thailand, from June to August 2021. The shallot bulb (*Allium ascalonicum* L.), licorice root (*Glycyrrhiza glabra* L.), and corn kernels (*Zea mays* L.) were purchased from a regular market in Chiang Mai, Thailand, between October and December 2021. Each plant was identified by the Pharmaceutical and Natural Products Research and Development Unit (PNPRDU), Chiang Mai University, Chiang Mai, Thailand, with the code numbers PNPRDU65007, PNPRDU65008, PNPRDU65009, and PNPRDU65010, respectively. Supercritical carbon dioxide (ScCO_2_) was used to extract the BB3-CMU rice bran and shallot bulb according to our previous study. In short, the samples were mixed with a co-solvent and added into the extraction column of the ScCO_2_ machine (Applied Separations, Allentown, PA, USA) [28]. At the same time, the corn kernels and licorice root were macerated (CE). The extraction conditions of each plant, including the type of co-solvents, the ratios between plant material and solvent, pressure, temperature, and time, are shown in Table 4. After the extraction, the extract solution was filtered through Whatman filter paper no. 1 and evaporated at 50 °C by an evaporator until completely dried [32,74]. Moreover, guava (*Psidium guajava* L.) extract and the bran extracts of *Oryza sativa* cv. Bue Bang 4-CMU (BB4-CMU), KDML105, and Pieisu 1-CMU were derived from our previous study [2,17,75].

### 3.3. Phytochemical Evaluations of Plant Extract

#### 3.3.1. Total Phenolic Content

The total phenolic content of plant extract was measured following the method described by Ruksiriwanich et al., with minor adjustments. In summary, the extract was reacted with 10% (*v*/*v*) Folin–Ciocalteu reagent in a microtiter plate. Afterward, 6% (*w/v*) sodium bicarbonate solution was added and incubated for 2 h. The absorbance was recorded at 765 nm using a UV–Vis spectrophotometer (EZ Read 400 Flexi, Biochrom, Cambridge, UK) [28]. Gallic acid (10–200 mg/mL) served as the standard, and results were reported as milligrams of gallic acid equivalents per gram of extract (mg GAE/g extract). All experiments were conducted in triplicate.

#### 3.3.2. Total Flavonoid Content

The total flavonoid content was measured following the method described by Ruksiriwanich et al., with minor adjustments [16]. Briefly, the plant extract was mixed with distilled water and 7.5 µL of a 5.0% (*w/v*) NaNO_2_ solution, respectively. The mixture solution was reacted for 5 min. Afterward, 10% (*w/v*) AlCl_3_·6H_2_O solution, 1 M NaOH, and distilled water were loaded into a mixture solution, respectively. Lastly, the absorbance was measured at 510 nm using a UV-Vis spectrophotometer. Epigallocatechin gallate (30–300 mg/mL) served as the standard, and results were reported as a milligram of epigallocatechin gallate equivalents per gram of extract (mg EGCG/g extract). All experiments were conducted in triplicate.

#### 3.3.3. Quantitative Analysis of Glycyrrhizic Acid, Phenolic, and Flavonoid Profiles by Liquid Chromatography-Quadrupole Time-of-Flight Mass Spectrometry

The phenolic, flavonoid, and glycyrrhizic acid of the plant extracts were characterized using LC-QTOF-MS, adapted from a previously used method [76]. The analysis was conducted using an Agilent 1290 Infinity II system coupled with a 6546 LC-QTOF instrument (Agilent Technologies, Santa Clara, CA, USA). Chromatographic separation was achieved on a ZORBAX Eclipse Plus C18 column (2.1 × 150 mm, 1.8 μm) over a 26 min run. The mobile phase comprised solvent A (acetonitrile) and solvent B (distilled water with 0.1% formic acid). A gradient elution started at 50% solvent A for 10 min, gradually increasing to 100% solvent B over 15 min, and held until the end of the run. The flow rate was maintained at 1000 µL/min with an injection volume of 10 μL. The instrument operated in positive electrospray ionization (ESI) mode, with the following settings: capillary voltage of 4500 V, nebulizer gas (N2) at 35 psi, flow rate of 8000 mL/min, and a dry heater temperature of 320 °C. Mass spectra were acquired in the *m*/*z* range of 50–1000. Compound identification was facilitated using the ChemSpider online database.

#### 3.3.4. Quantitative Analysis of γ-Oryzanol and Tocopherol by High Performance Liquid Chromatography (HPLC)

The analysis of *γ*-oryzanol and tocopherol was adapted from the method of Pestana-Bauer et al. [77]. For *γ*-oryzanol quantification, plant extracts were diluted in dichloromethane to a final concentration of 1000 µg/mL, then filtered through a 0.45 μm syringe filter. The plant extracts were analyzed using an HPLC machine (Shimadzu, Kyoto, Japan) with an Ultra C18 column (5 μm, 4.6 × 250 mm; Restek, PA, USA) and a Shimadzu UV–Vis detector coupled with an SPD-20A diode array detector. The mobile phase consisted of methanol, acetonitrile, dichloromethane, and acetic acid in a ratio of 50:44:3:3, with a flow rate of 1400 mL/min, and the detection wavelength was set at 330 nm.

For tocopherol analysis, a Shimadzu HPLC machine equipped with a fluorescence detector (RF-20A; Shimadzu Corporation, Kyoto, Japan) was used. The separation was carried out on a reverse-phase Ultra C18 column (5 μm, 250 × 4.6 mm). The mobile phase consisted of three mixtures: acetonitrile, methanol, and isopropanol in the ratios 50:40:10 (A) and 30:65:5 (B). The gradient started with 85% A for 15 min, decreased to 10% A over 2 min, then increased back to 50% over 5 min, and finally to 85% over 3 min. The flow rate was set at 1000 µL/min, with a total run time of 26 min. The fluorescence detector was set to an excitation wavelength of 290 nm and an emission wavelength of 330 nm.

#### 3.3.5. Determination of Antioxidant Activities

##### DPPH Radical Scavenging Assay

The DPPH radical-scavenging activity was evaluated as previously described [28]. In brief, the plant extract was loaded into a microtiter plate, followed by distilled water and a 0.1 mM ethanolic DPPH solution. After that, the plate was stored in the dark for 30 min. The absorbance of the mixture solution was read at a wavelength of 515 nm using the UV–Vis spectrophotometer. Trolox was employed as a standard scavenger, and 95% (*v*/*v*) ethanol was used as a blank in these experiments. The DPPH radical scavenging activity was calculated using Equation (1):

(1) $$DPPH\;radical\;scavenging\;activity\;\left(\%\right)=\frac{\left({Optical\;density\;}_{control}-{Optical\;density\;}_{sample}\right)}{{Optical\;density\;}_{control}}\times 100$$

where ${Optical\;density\;}_{control}$ absorbance of the DPPH solution mixture and ethanol, and ${Optical\;density}_{\;sample}$ = absorbance of the DPPH radical solution mixture and sample.

##### ABTS Radical Scavenging Assay

The ABTS assay was performed as previously described [2]. In short, the ABTS stock solution was prepared in equal amounts of two stock solutions (7.0 mM ABTS solution and 2.45 mM potassium persulfate solution). The mixture was stored in the dark for 12–16 h to react. After that, the ABTS stock solution was diluted with 95% (*v*/*v*) ethanol to acquire an absorbance of 0.70 ± 0.02 units 734 nm using a UV–Vis spectrophotometer. The ABTS working solution was prepared daily. Then, the plant extract and ABTS working solution were loaded into a microtiter plate and kept in the dark for 30 min. The absorbance of the mixture solution was recorded at 734 nm. Trolox was used as the standard, and 95% (*v*/*v*) ethanol served as the blank. The ABTS radical scavenging activity was calculated using Equation (2):

(2) $$ABTS\;radical\;scavenging\;activity\;\left(\%\right)=\frac{\left({Optical\;density}_{\;control}-{Optical\;density}_{\;sample}\right)}{{Optical\;density}_{\;control}}\times 100$$

where $Optical\;density_{\;control}$ absorbance of the ABTS solution mixture and ethanol, and ${Optical\;density\;}_{sample}$ = absorbance of the ABTS radical solution mixture and sample.

### 3.4. Preparation of Hair Rise^TM^ Microemulsion

A Hair Rise^TM^ microemulsion for hair growth promotion and anti-hair loss was prepared in three phases (A–C). First, the ingredients of the oil based phase (A), including BB3-CMU rice bran extract, BB4-CMU rice bran extract [17], KDML105 rice bran extract [2], Piesu rice bran extract [75], shallot extract [16], and licorice root extract, were weighted and dissolved in Tween 80, Tween 20, and PEG-40 hydrogenated castor oil as co-solvents. For the water-based phase (B), vitamin B5, guava extract [24], and disodium EDTA were dissolved in distilled water. Finally, for the propylene glycol-based phase (C), corn kernel extract was dissolved in propylene glycol.

For the preparation of Hair Rise^TM^ microemulsion according to the drug delivery system (low energy), phase A was heated in a magnetic stirrer set to 40 °C at 300 rpm. Phase B was added to the A phase, maintaining continuous stirring for 10 min. After that, the C phase was added and mixed until fully dissolved. The prepared Hair Rise^TM^ microemulsion was removed from the hot plate magnetic stirrer and transferred to a container for cooling. The composition of the Hair Rise^TM^ microemulsion is shown in Table 5.

### 3.5. Measurement of Particle Size, Polydispersity Index, and Zeta Potential Values of Hair Rise^TM^ Formulation

The particle size, or Z-average (Z-Ave), polydispersity index (pdI), and zeta potential (ZP) value of Hair Rise^TM^ microemulsion were determined using Zetasizer Nano ZS with a dynamic light scattering technique (Malvern Panalytical, Bristol, UK). The physical stability of Hair Rise^TM^ microemulsion was evaluated by measuring Z-Ave, pdI, and ZP after storing the microemulsion at 30 °C for 3 months, with assessments at initial, 1, 2, and 3 months. The heating–cooling cycle test was conducted to assess the stability of Hair Rise^TM^ microemulsion under extreme temperature variations, such as during transportation. In this test, the microemulsion was alternately stored at 4 °C and 45 °C for 24 hours per temperature (one cycle), and this process was repeated for 6 cycles. All of the samples were evaluated using integrated dynamic light scattering (DLS) for 1 min. The Malvern’s Dispersion Technology Software (DTS) version 7.13 was used to calculate the DLS data [78]. 

### 3.6. In Vitro Cell Viability Assay

The primary human hair follicle dermal papilla cells (HFDPCs) were obtained from Promo Cell GmbH (Heidelberg, Germany) and cultured using the Dermal Papilla Growth Medium, supplemented with a 1% antibiotic-antimycotic solution. Furthermore, human prostate cancer cells (DU-145) and macrophage cells (RAW 264.7) were sourced from the American Type Culture Collection (Rockville, MD, USA). DU-145 cells were maintained in RPMI-1640 culture medium with the addition of 10% FBS and 1% penicillin/streptomycin solution. RAW 264.7 cells were maintained in DMEM culture medium, also supplemented with 10% FBS and 1% penicillin/streptomycin solution.

The sulphorhodamine B (SRB) method was used to assess the viability and proliferation of cells treated with plant extracts, Hair Rise^TM^ microemulsion at varying concentrations (50, 75, and 100% *v*/*v* in water), and standard controls (including diclofenac sodium, dutasteride, finasteride, and minoxidil) at concentrations ranging from 0.0625 to 2 mg/mL in RAW 264.7, DU-145, and HFDPCs [79]. Cells were added into 96-well plates at a density of 1 × 10^5^ cells/mL and incubated for 24 h at 37 °C with 5% CO_2_. Then, the cells were treated with the samples and exposed to all treatments for 24 h. Afterward, the monolayer cells were fixed with 50% (*w/v*) trichloroacetic acid at 4 °C for 1 h, followed by staining with 0.04% (*w/v*) SRB solution for 30 min. Lastly, the bound dye was removed by 10 mM Tris base, and the absorbance was read at 515 nm. The concentrations that provided more than 80% cell viability were collected for further experiments. Cell viability was calculated using Equation (3).

(3) $$Cell\;viability\;\left(\%\right)=\frac{\left({Optical\;density}_{\;sample}-{Optical\;density\;}_{blank}\right)}{\left({Optical\;density}_{\;control}-{Optical\;density\;}_{blank}\right)}\times 100$$

where ${Optical\;density}_{\;sample}$ is the absorbance of the cells treated with plant extracts, Hair Rise^TM^ microemulsions or the standard controls, ${Optical\;density\;}_{control}$ is the absorbance of the cells treated with ethanol in culture medium, and ${Optical\;density\;}_{blank}$ is the absorbance of the 96-well plate.

### 3.7. Anti-Inflammatory Activity Assay

The quantification of nitrite that increased in the cell culture supernatants was evaluated by the Griess reaction colorimetric assay kit. In short, the HFDPCs and RAW 264.7 cells were seeded (1 × 10^5^ cells/mL) and incubated for 24 h. Afterward, the cells were pretreated with 0.5 mg/mL of standard controls (diclofenac sodium), Hair Rise^TM^ microemulsion in different concentrations (50, 75, and 100% (*v*/*v*) in water), and incomplete medium (blank) for 2 h. Afterward, the cells were exposed to 0.001 mg/mL LPS for 24 h. Cell culture supernatants were collected and reacted with the Griess reagent according to the manufacturer’s instructions. A calibration curve was generated from standard nitrite at a concentration of 0.01 to 100 µM [76].

### 3.8. Thiobarbituric Acid-Reactive Substances (TBARS) Assay

The antioxidant activity of standard controls (L-ascorbic acid), Hair Rise^TM^ microemulsion in different concentrations (50, 75, and 100% (*v*/*v*) in water), and the control group (incomplete medium) was determined using the TBARS method. In brief, HFDPCs were added to 6-well plates (1 × 10^5^ cells/mL) and incubated for 24 h. Subsequently, the cells were pretreated with 0.5 mg/mL of each sample for 24 h, followed by post-treatment with H_2_O_2_ for 2 h. The cells were harvested and reacted with a mixture solution (1% Triton X-100, 0.6% thiobarbituric acid, and 15% trichloroacetic acid) at 100 °C for 10 min. Afterward, the cells were cooled down in a −80 °C freezer for 10 min. The final product of lipid peroxidation was read at 532 nm. The quantification of malondialdehyde in HFDPCs was determined in comparison to the untreated cells (control group) [76].

### 3.9. Semi-Quantitative Reverse Transcription and Polymerase Chain Reaction Analysis

The expression of the gene in the androgen pathway (*SRD5A1-3*) and the genes associated with hair growth, including Wnt/β-catenin (*CTNNB1*), Sonic Hedgehog (*SHH*, *SMO*, and *GLI1*), and angiogenesis pathways (*VEGF*), was evaluated in DU-145 and HFDPCs as previously established [28]. Hair Rise^TM^ microemulsion at different concentrations (50, 75, and 100% (*v*/*v*) in water) was compared to the standard controls (dutasteride, finasteride, and minoxidil) at 0.5 mg/mL. RNA was extracted using the E.Z.N.A.^®^ Total RNA Kit I (Omega BioTek, Norcross, GA, USA). The RNA concentration was quantified utilizing the Qubit^TM^ RNA HS Assay Kit alongside the Qubit^TM^ 4 fluorometer (Invitrogen, Carlsbad, CA, USA). Gene expression analysis was carried out with the MyTaq^TM^ One-Step RT-PCR Kit (Bioline, Memphis, TN, USA), and the specific primer sequences used are listed in Table 6. Glyceraldehyde 3-phosphate dehydrogenase (*GAPDH*) was used as the reference gene. The results were shown as fold changes in gene expression. PCR products were visualized by agarose gel electrophoresis, with images and band intensity analyzed using the Gel Doc^TM^ EZ System and Image Lab^TM^ software 5.1 (Bio-Rad Laboratories, Hercules, CA, USA).

### 3.10. Statistical Analysis

The results are expressed as mean ± standard deviation (SD). Statistical analyses were performed using one-way ANOVA, followed by Tukey’s post hoc test, with significance defined at *p* < 0.05. Data processing and analysis were conducted using statistical software (version 23.0).

## 4. Conclusions

A green cosmeceutical Hair Rise^TM^ microemulsion containing medicinal extracts from ScCO_2_ BB3-CMU rice bran, BB4-CMU rice bran, KDML105 rice bran, Piesu 1 CMU rice bran, guava leaf, shallot bulb, licorice root, and corn kernels was successfully formulated to promote hair growth and combat hair loss. Hair Rise^TM^ microemulsion at all concentrations (50%, 75%, and 100% *v*/*v* in water) promoted cell proliferation of HFDPCs, particularly during the anagen phase of the hair cycle. They also demonstrated anti-inflammatory and antioxidative properties by suppressing nitric oxide and TBARS levels, which reduced inflammation and oxidative damage in hair follicles. In addition, all concentrations of Hair Rise^TM^ microemulsion showed significantly higher inhibitory effects on *SRD5A* expression than minoxidil, finasteride, and dutasteride, which could decrease the synthesis of androgenic hormone in hair follicles and present an anti-hair loss effect. Furthermore, all concentrations of Hair Rise^TM^ microemulsion stimulated the expression of hair growth factor genes, including *CTNNB1*, *SHH*, *SMO*, *GLI1*, and *VEGF*, to support hair growth by extending the anagen phase and enhancing blood flow to the hair follicles. In summary, our findings suggest that Hair Rise^TM^ microemulsion at all concentrations, particularly the 100% formulation, represents the most effective treatment for AGA, highlighting the potential of green cosmetics in promoting hair growth and minimizing hair loss. However, a further efficacy test of Hair Rise^TM^ microemulsion should be arranged in AGA patients. 

## Figures and Tables

**Table 1:** Bioactive compounds and antioxidant activities of plant extracts.

Results		Plant Extract			
		BB3-CMU Rice Bran	Shallot Bulb	Licorice Root	Corn Kernels
Extraction yield (%)		14.80 ± 0.04	5.85 ± 0.09	11.21 ± 0.24	22.41 ± 0.86
Total phenolic content (mg GAE/g extract)		32.42 ± 1.42	4.18 ± 0.03	3.21 ± 0.12	14.42 ± 0.56
Total flavonoid content (mg EGCG/g extract)		56.03 ± 1.14	2.54 ± 0.01	1.96 ± 0.08	4.33 ± 0.12
Chlorogenic acid (mg/g extract)		0.11 ± 0.01	-	0.12 ± 0.01	4.22 ± 0.51
*p*-Coumaric acid (mg/g extract)		0.07 ± 0.01	1.02 ± 0.01	-	0.33 ± 0.01
Rosmarinic acid (mg/g extract)		0.05 ± 0.01	0.21 ± 0.01	-	-
Ferulic acid (mg/g extract)		0.02 ± 0.01	-	-	5.14 ± 0.01
Epigallocatechin gallate (mg/g extract)		0.03 ± 0.03	-	0.09 ± 0.02	-
Quercetin (mg/g extract)		0.03 ± 0.01	0.03 ± 0.01	0.07 ± 0.01	-
*α*-Tocopherol (mg/g extract)		8.54 ± 0.02	-	-	-
*γ*-Tocopherol (mg/g extract)		3.65 ± 0.01	-	-	-
Glycyrrhizic acid (mg/g extract)		-	-	0.62 ± 0.02	-
Antioxidant activities (%)	ABTS	32.14 ± 2.03	21.16 ± 1.33	16.15 ± 0.21	14.36 ± 0.04
	DPPH	28.89 ± 1.08	19.15 ± 0.96	14.16 ± 0.24	12.18 ± 0.06

Note: Values are presented as the mean ± standard deviation (SD). Milligrams of gallic acid equivalents per gram of extract (mg GAE/g extract); milligrams of epigallocatechin gallate equivalents per gram of extract (mg EGCG/g extract); milligrams per gram of extract (mg/g extract).

**Table 2:** Physical stability of Hair Rise^TM^ microemulsion after storage for 3 months and six cycles of heating–cooling acceleration.

Condition	Size (nm)	Zeta Potential (mV)	pdI
At initial	30.57 ± 0.17 ^a^	−3.36 ± 0.05 ^a^	0.23 ± 0.02 ^b^
After 1 month	32.43 ± 0.06 ^a^	−1.87 ± 0.03 ^b^	0.11 ± 0.01 ^a^
After 2 months	38.96 ± 1.32 ^b^	−0.27 ± 0.01 ^e^	0.26 ± 0.02 ^c^
After 3 months	48.38 ± 0.88 ^d^	−0.34 ± 0.02 ^d^	0.21 ± 0.03 ^b^
After six cycles of heating–cooling accelerate process	42.43 ± 0.91 ^c^	−0.66 ± 0.02 ^c^	0.23 ± 0.03 ^b^

Note: Values are presented as the mean ± SD for triplicates in each sample. Statistical analyses were performed using one-way ANOVA, followed by Tukey’s HSD test, with significance defined at *p* < 0.05 in each sample. Statistical differences are represented by different letters (a–e).

**Table 4:** Extraction methods and the extraction conditions of plant material extracts.

Plants Materials	Extraction Method	Co-Solvent	Ratio (Sample: Co-Solvent)	Pressure (Bar)	Temperature (°C)	Time (h)
BB3-CMU rice bran	ScCO_2_	95% (*v*/*v*) Ethanol	1:1	400	50	0.5
Shallot bulb	ScCO_2_	50% (*v*/*v*) Ethanol	2:1	400	50	0.5
Licorice root	CE	30% (*v*/*v*) Ethanol	1:2	-	25	24
Corn kernels	CE	50% (*v*/*v*) Ethanol	1:2	-	25	24

**Table 5:** Compositions of Hair Rise^TM^ microemulsion (*w*/*w*).

Phase	Ingredient	Quantity % (*w*/*w*)
A	BB3-CMU rice bran extract	1
	BB4-CMU rice bran extract	1
	KDML105 rice bran extract	1
	Piesu 1 CMU rice bran extract	1
	Tween 80	8.5
	Tween 20	2
	PEG-40 hydrogenated castor oil	10
B	Vitamin B5	1
	Guava extract	2
	Shallot extract	1
	Licorice root extract	1.5
	Disodium EDTA	0.5
	Distilled water	56.5
C	Corn kernels extract	3
	Propylene glycol	10

**Table 6:** Specific primer sequences used for semi-quantitative RT-PCR.

Functional Pathway	Gene Name	Gene Bank No.	Primers	Sequence (5′-3′)
Androgen	*SRD5A1*	NM_001047.4	Forward Reverse	AGCCATTGTGCAGTGTATGC AGCCTCCCCTTGGTATTTTG
	*SRD5A2*	NM_000348.4	Forward Reverse	TGAATACCCTGATGGGTGG CAAGCCACCTTGTGGAATC
	*SRD5A3*	NM_024592.5	Forward Reverse	TCCTTCTTTGCCCAAACATC TCCTTCTTTGCCCAAACATC
Wnt/β-catenin	*CTNNB1*	NM_001330729.2	Forward Reverse	CCCACTAATGTCCAGCGTTT AACCAAGCATTTTCACCAGG
Sonic Hedgehog	*SHH*	NM_000193.4	Forward Reverse	AAAAGCTGACCCCTTTAGCC GCTCCGGTGTTTTCTTCATC
	*SMO*	NM_005631.5	Forward Reverse	GAAGTGCCCTTGGTTCGGACA CCGCCAGTCAGCCACGAAT
	*GLI1*	NM_005269.3	Forward Reverse	GCAGGGAGTGCAGCCAATACAG GAGCGGCGGCTGACAGTATA
Angiogenesis	*VEGF*	NM_001025366.3	Forward Reverse	CTACCTCCACCATGCCAAGT GCGAGTCTGTGTTTTTGCAG
Internal control	*GAPDH*	NM_001289745.3	Forward Reverse	GGAAGGTGAAGGTCGGAGTC CTCAGCCTTGACGGTGCCATG

## Data Availability

Data are contained within the article and Supplementary Materials.

## Supplementary Materials

The following supporting information can be downloaded at: https://www.mdpi.com/article/10.3390/plants13192802/s1, Table S1: The effects of different proportions of plant extracts on the gene expression of SRD5A2 in DU-145 cells.
