# Supplementary material for: Synergistic Phytochemical and Pharmacological Actions of Hair RiseTM Microemulsion: A Novel Herbal Formulation for Androgenetic Alopecia and Hair Growth Stimulation
Source: Plants (Basel). 2024 Oct 6;13(19):2802. doi: 10.3390/plants13192802 (PMC11479085; doi:10.3390/plants13192802)
Supplement: Supplementary file 1 [file plants-13-02802-s001.zip › plants-3195717-supplementary.pdf]

**Proportions of Plant Extracts in Hair Rise™ microemulsion**

The optimal ratio of the extracts (rice bran: guava leaf: licorice root: corn kernel) at 1:2:2:3 was selected, as shown in **Table S1.** This proportion was from our preliminary study for 3 rounds to investigate the best proportion that showed the highest *SRD5A2* inhibition activity, which gave the lowest fold changes. In the 1st round, proportions 3, 4, and 5 were selected for the 2nd round. In the 2nd round, the proportion 2 was selected. In the 3rd round, proportion 1 was selected for Hair Rise™ microemulsion.

**Table S1.** The effects of different proportions of plant extracts on the gene expression of *SRD5A2* in DU-145 cells.

| **Round 1** | | | **Round 2** | | | **Round 3** | | |
|---|---|---|---|---|---|---|---|---|
| **No.** | **Proportions** (RB: GL: LR: CK) | ***SRD5A2*** **(Fold change)** | **No.** | **Proportions** (RB: GL: LR: CK) | ***SRD5A2*** **(Fold change)** | **No.** | **Proportions** (RB: GL: LR: CK) | ***SRD5A2*** **(Fold change)** |
| 1 | 1: 1: 1: 1 | 0.66 ± 0.01 | 1 | 1: 2: 2: 1 | 0.53 ± 0.01 | 1 | 1: 2: 2: 3 | 0.22 ± 0.01 |
| 2 | 2: 1: 1: 1 | 0.71 ± 0.02 | 2 | 1: 2: 2: 2 | 0.31 ± 0.02 | 2 | 1: 2: 2: 4 | 0.28 ± 0.02 |
| 3 | 1: 2: 1: 1 | 0.54 ± 0.02 | 3 | 1: 3: 3: 1 | 0.48 ± 0.01 | | | |
| 4 | 1: 1: 2: 1 | 0.58 ± 0.01 | 4 | 1: 3: 3: 2 | 0.42 ± 0.01 | | | |
| 5 | 1: 1: 1: 2 | 0.52 ± 0.01 | | | | | | |

**Note:** RB: Rice bran extract; GL: Guava leaf; LR: Licorice root and CK: Corn kernels
